# Clinical efficacy of Fufang Yinhua Jiedu (FFYH) granules in mild COVID-19 and its anti-SARS-CoV-2 mechanism by blocking autophagy through inhibiting the AKT/mTOR signaling pathway

**DOI:** 10.3389/fphar.2024.1431617

**Published:** 2024-09-16

**Authors:** Wenlei Wang, Zhihui Zheng, Xiaoyuan Qi, Hailin Wei, Xuhua Mao, Qin Su, Xiang Chen, Yan Feng, Guohong Qiao, Tieliang Ma, Zhian Tang, Guangming Zhou, Jinqiang Zhuang, Pinghu Zhang

**Affiliations:** ^1^ Jiangsu Key Laboratory of Integrated Traditional Chinese and Western Medicine for Prevention and Treatment of Senile Diseases, Medical College, Yangzhou University, Yangzhou, China; ^2^ National Human Diseases Animal Model Resource Center, NHC Key Laboratory of Human Disease Comparative Medicine, National Center of Technology Innovation for Animal Model, Institute of Laboratory Animal Sciences, Chinese Academy of Medical Science and Peking Union Medical School, Beijing, China; ^3^ Yixing People’s Hospital, Yixing, Jiangsu, China; ^4^ Emergency Intensive Care Unit (EICU), Affiliated Hospital of Yangzhou University, Yangzhou University, Yangzhou, Jiangsu, China; ^5^ Jiangsu Key Laboratory of Zoonosis, Jiangsu Co-Innovation Center for Prevention and Control of Important Animal Infectious Diseases and Zoonoses, Yangzhou University, Yangzhou, Jiangsu, China

**Keywords:** COVID-19, Fufang Yinhua Jiedu granules, PI3K/AKT/mTOR, cytokine storm, autophagy

## Abstract

**Background:**

Fufang Yinhua Jiedu (FFYH) granules are recommended for treating coronavirus pneumonia (COVID-19) in China. However, its anti-severe acute respiratory syndrome coronavirus 2 (SARS-CoV-2) activity and clinical efficacy against COVID-19 remain to be confirmed.

**Aims:**

Our study aimed to investigate the anti-SARS-CoV-2 effect and potential mechanism of FFYH.

**Materials and Methods:**

The activity of FFYH against severe acute respiratory syndrome coronavirus 2 (SARS-CoV-2) was evaluated via cell pathogenic effects, immunoblotting, immunofluorescence staining, and qRT-PCR. The potential mechanism of FFYH against SARS-CoV-2 was investigated by immunoblotting. One head-to-head randomized controlled trial was designed to evaluate the clinical efficacy of FFYH in mild COVID-19. Two hundred patients were randomly recruited to receive either FFYH or LHQW (Lianhua Qingwen) granules.

**Results:**

The *in vitro* results indicated that FFYH effectively inhibited SARS-CoV-2 replication by suppressing CPE and decreasing viral RNA and protein expression. A time-of-drug-addition assay confirmed that FFYH mainly targeted the binding and replication stages of the SARS-CoV-2 life cycle. Mechanistic studies revealed that blocking SARS-CoV-2-triggered autophagy may be the primary mechanism by which FFYH protects against SARS-CoV-2 infection by regulating the phosphatidylinositol 3-kinase (PI3K)/AKT/mammalian target of rapamycin (mTOR) signaling pathway. Clinical results confirmed that FFYH effectively shortened the recovery time of clinical symptoms and viral nucleic acid negativity, improved abnormal hematology parameters, and controlled excessive cytokine responses in mild COVID-19 patients. Subgroup analysis revealed that FFYH improved the recovery time of clinical symptoms, improved hematological parameters, and controlled excessive cytokine storms to a greater extent in the mild COVID-19 male subgroup, abnormal hematology subgroup, and 32–42-year-old subgroup than in the corresponding LHQW subgroup (*P* < 0.05). No patients progressed to severe or critical cases.

**Conclusion:**

Our results indicate that FFYH not only has good anti-viral activity against SARS-CoV-2 but also has significant efficacy against COVID-19, indicating that FFYH may be a novel complementary option for treating COVID-19.

## Introduction

Since the first case of novel coronavirus pneumonia (COVID-19) was diagnosed, severe acute respiratory syndrome coronavirus 2 (SARS-CoV-2) has caused countless infections and more than 7,000,000 deaths. Most patients have a good prognosis, but some patients progress to critical cases or die. Most critical patients are only presented with mild fever, cough, or muscle soreness at the initial stages. However, some patients suddenly and rapidly progress to acute respiratory distress syndrome (ARDS) or multiple organ failure (Chen et al., 2020; Huang et al., 2020). It has been reported that excessive cytokines in patients are strongly associated with the process of disease aggravation (Karki et al., 2021; Yang et al., 2021). Many studies have demonstrated that timely control of excessive cytokine storms can improve the prognosis of severe cases, suggesting that regulating excessive inflammatory responses may be an effective strategy for the treatment of COVID-19 (Zhang et al., 2022b). However, given that anti-viral therapies have limited effects on cytokine responses, novel therapies or drugs are urgently needed to control excessive inflammatory responses caused by SARS-CoV-2 effectively (Karki et al., 2021; Yang et al., 2021; Kim et al., 2021).

Traditional Chinese Medicine (TCM) has been used to treat respiratory infectious diseases in China. Several clinical studies have reported that TCM is effective in improving the clinical symptoms of COVID-19 (Zhang et al., 2022a). Fufang Yinhua Jiedu (FFYH) granules is a Chinese patent medicine composed of *Artemisia annua* L (Asteraceae; Artemisia annua herba)*, Lonicerae japonica* Thunb (Caprifoliaceae; Lonicerae japonica Flos), *Schizonepeta tenuifolia* (Benth.) Briq (Lamiaceae; *Schizonepeta* herba)*, Mentha canadensis* L (Lamiaceae; Menthae haplocalycis herba), *Chrysanthemum indicum* L (Asteraceae; Chrysanthemi indici Flos), *Isatis tinctoria* L (Brassicaceae; Isatidis radix), *Forsythia suspensa* (Thunb.) Vahl (Oleaceae; Forsythiae fructus), *Commelina communis* L (Commelinaceae; Commelinae herba), *Peucedanum praeruptorum* Dunn (Apiaceae; Peucedani radix), and *Glycine* max (L.)Merr (Fabaceae; Sojae semen praeparatum). FFYH has been approved by the Chinese Food and Drug Administration for treating upper respiratory tract infectious diseases such as influenza. Our previous work demonstrated that FFYH exhibited a good protective effect on influenza virus infection (Zhang et al., 2021). Our recent work also confirmed that FFYH had good anti-viral activity against HCoV-OC43 and HCoV-229E (Zheng et al., 2022). However, whether FFYH is effective for treating COVID-19 remains unclear. Here, the anti-SARS-CoV-2 activity and clinical efficacy of FFYH for mild COVID-19 patients were evaluated, and the potential mechanism by which FFYH affects SARS-CoV-2 was elucidated.

## Materials and methods

### Reagents

Dulbecco’s modified Eagle’s medium/F12 (DMEM/F12) (C11330500BT) and fetal bovine serum (2413620P) were purchased from Gibco (Gaithersburg, MD). Trypsin (C100C1) was purchased from Kaiji (Nanjing, China). Penicillin and streptomycin solution (03-031-1B) was purchased from Biological Industries (Beit HaEmek, Israel). AKT (AA326) antibodies and β-actin (AA128) antibodies were obtained from Beyotime (Shanghai, China). Phospho-Akt (Ser473, #4060), mTOR (7C10, #2983), phospho-mTOR (Ser2448, #5536), phospho-mTOR (Ser2481, #2974), phospho-p70S6 kinase (Ser371, #9208), phospho-p70S6 kinase (Thr389, #9205), LC3A/B (12741P), and SQSTM1/P62 (8025S) antibodies were purchased from Cell Signaling Technology (CA, USA). The HiScript II 1st Strand cDNA Synthesis Kit (R222) and AceQ Universal SYBR qPCR Master Mix (Q511) were purchased from Vazyme (Nanjing, China). CQ (c6628) and BafA1 (B1793) were obtained from Sigma. FFYH was provided by Yifan Pharmaceutical (Hangzhou, China) and was prepared according to the method described in Supplementary Material S2. Lianhua Qingwen capsules or granules produced by Shijiazhuang Yiling Pharmaceutical (Shijiazhuang, China) were selected as positive drug controls because of their ability to treat COVID-19 effectively. Molnupiravir, a positive anti-viral control, was obtained from Selleck Chemicals (Shanghai, China).

### Virus and cells

The Vero E6 cell line was purchased from the China Center for Type Culture Collection (CCTCC) and was used to determine the virus titer of SARS-CoV-2. A549-ACE2 cells were provided by Prof. Hongqi Liu from the Institute of Laboratory Animal Science (CAM&PUMC) and were used to assess the anti-viral effect and mechanism of action of FFYH. The cells were cultured with the DMEM/F12 medium supplemented with 10% fetal bovine serum and 100 U/mL penicillin or streptomycin. The 50% tissue culture infective dose (TCID_50_) of the virus was determined as previously described (Zhang et al., 2021).

### Cytotoxicity assay

As previously described (Zhang et al. 2021), an MTT assay was conducted to investigate the cytotoxicity of FFYH.

### Anti-viral activity assay

The anti-viral effect of FFYH against SARS-CoV-2 was determined as previously described (Wei et al., 2024). In brief, A549-ACE2 cells were seeded into 96-well plates at 4×10^4^ cells/well overnight and then infected with 100 TCID_50_ viruses (0.01 multiplicity of infection (MOI) diluted with 50 μL of PBS). After infection for 1 h, the plates were incubated with the indicated drugs for 72 h. The protective effect of FFYH on the pathogenic effect of SARS-CoV-2 was observed under an inverted microscope.

### Immunofluorescence analysis

Immunofluorescence analysis was performed as previously described (Wei et al., 2024). In brief, A549-ACE2 cells were infected and treated as described for the anti-viral activity assay. Then, 24 h after infection, the plates were fixed with 4% paraformaldehyde for 2 h, exposed to primary mouse anti-SARS-CoV-2 NP antibody, and then incubated with the FITC-labeled anti-mouse IgG secondary antibody. The anti-viral effects of FFYH were recorded with a fluorescence microscope (Leica DMI3000B, Germany).

#### Time-of-drug-addition assay

**FIGURE 1:**
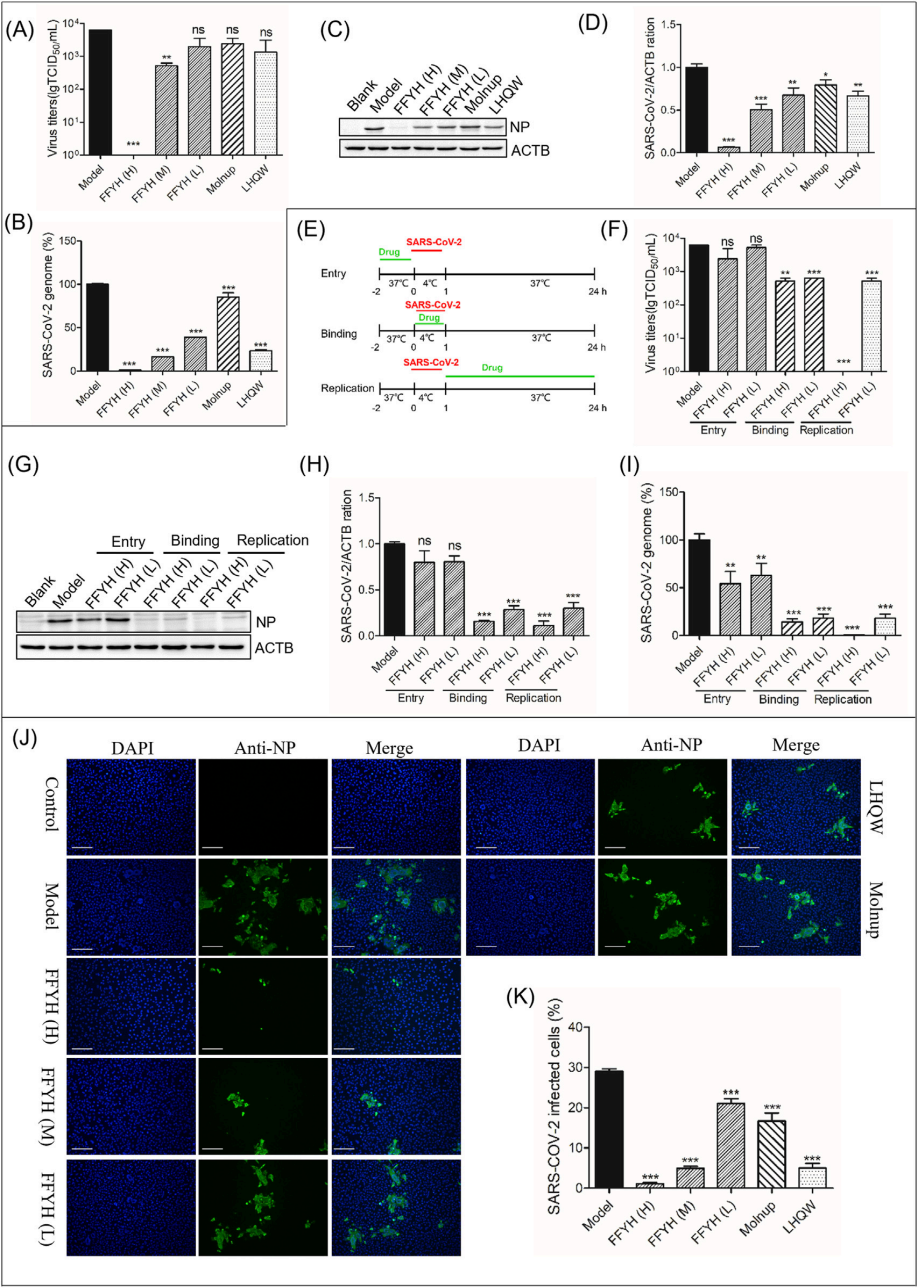
Anti-viral efficacy of FFYH against SARS-CoV-2. FFYH dose-dependently inhibited the production of newborn SARS-CoV-2 **(A)**, SARS-CoV-2 viral RNA transcription **(B)**, and SARS-CoV-2 viral protein expression **(C, D)**. Schematic diagram of the time-of-drug-addition assay for FFYH **(E)**. Effects of FFYH on virus binding, entry, and replication were confirmed by a viral titer assay **(F)**, protein expression levels **(G, H)**, and viral RNA levels **(I)**. Immunofluorescence assays further verified the anti-SARS-CoV-2 effect of FFYH **(J, K)**. FFYH **(H)** 1,000 μg/mL and FFYH (L) 500 μg/mL are expressed in Figures 1E–I. FFYH **(H)** 1,000 μg/mL, FFYH (M) 500 μg/mL, FFYH (L) 250 μg/mL; Molnupiravir, 2 μM; LHQW, 500 μg/mL in the other groups. ^ns^ *P*>0.05, ^*^ *P* < 0.05, ^**^ *P* < 0.01, and ^***^ *P* < 0.001 vs. the virus-infected group.

A time-of-drug-addition assay was performed as previously described (Wei et al., 2024). A549-ACE2 cells were infected at an MOI of 0.01 and then treated according to the methods of Figure 1E. The viral load of the cell supernatant was determined via a TCID_50_ assay, and the cells were lysed for immunoblotting analysis.

### Immunoblotting analysis

Western blotting was performed as previously described (Zhang et al., 2021). In brief, A549-ACE2 cells were infected at an MOI of 0.01 and then treated with the indicated drugs for 24 h. Cell lysates were separated via 6% or 12% SDS‒PAGE and then transferred onto NC membranes. After blocking with 5% BSA, the membrane was incubated with primary antibodies and then exposed to specific anti-mouse or anti-rabbit secondary antibodies. The protein bands were recorded with a Molecular Imager SH-523 System (Shenhua, Hangzhou).

### qRT-PCR analysis

qRT-PCR analysis was performed as previously described (Zhang et al., 2021). The relative levels of viral RNA were determined via the 2^−ΔΔCt^ calculation method, with GAPDH serving as an internal reference for normalization. The primers used to target the SARS-CoV-2 nucleocapsid protein (NP) were previously described (5′-TAA​TCA​GAC​AAG​GAA​CTG​ATT​A-3′ and 5′-CGA​AGG​TGT​GAC​TTC​CAT​G-3′) (Aliyari et al., 2022).

### Hematology parameter analysis

Blood samples were collected on day 1 and day 5 or 6. Hematology parameters were examined with a five-classification blood cell counter (Mindray (BC7500), China).

### Serum cytokine assays

The levels of 34 cytokines and chemokines in the serum were measured simultaneously via a Luminex assay via the Cytokine and Chemokine 34-Plex Human ProcartaPlex Panel (Invitrogen, EPX340-12167-901). The assay was performed and analyzed independently by Shanghai Laizee Biotech (Shanghai, China) via a Luminex 200 instrument and ProcartaPlex Analyst 1.0 software. In brief, 25 µL of serum from each patient was incubated for 2 h with a mixture of color-coded beads precoated with analyte-specific capture antibodies. After washing, biotinylated detection antibodies specific to the analytes of interest were added and formed an antibody-antigen sandwich, and then, phycoerythrin (PE)-conjugated streptavidin was added to bind the biotinylated detection antibodies. The beads were read on a Luminex 200 analyzer. One laser was used to classify the bead and determine the detected analyte. The second laser was used to determine the magnitude of the PE-derived signal, which was directly proportional to the amount of analyte bound. Standard curves for all 34 analytes were generated, and the concentration level of each analyte was determined via ProcartaPlex Analyst 1.0 software. Thirty-four cytokines and chemokines include interferons (IFN-α and IFN-γ), interleukins (IL-12 (p70), IL-13, IL-1β, IL-2, IL-4, IL-5, IL-6, IL-18, IL-10, IL-17A, IL-21, IL-22, IL-23, IL-27, IL-8, IL-9, IL-31, IL-15, IL-1α, IL-1RA, and IL-7), tumor necrosis factor (TNF-α and TNF-β), granulocyte-macrophage colony-stimulating factor (GM-CSF), epsilon, chemokine growth-related oncogene-alpha (GRO-α), interferon-gamma inducible protein-10 (IP-10), monocyte chemoattractant protein-1 (MCP-1), macrophage-inflammatory protein (MIP-1α and MIP-1β), and stromal cell-derived factor-1α (SDF-1α) and are regulated upon activation of normal T cell expressed and secreted (RANTES).

#### Study design

One randomized, single-blind, controlled trial was designed to evaluate the efficacy of FFYH in mild COVID-19 patients (Figure 3A). The trial protocol is available at ClinicalTrial.gov (ChiCTR2200066613). The trial was performed according to the principles of the Declaration of Helsinki and the Good Clinical Practices and Chinese regulations. This trial was conducted at Yixing People’s Hospital. The Ethics Committee of Yixing People’s Hospital approved this trial (LS 2022K172-01), and all participants signed informed consent. This trial followed the Consolidated Standards of Reporting Trials (CONSORT) reporting guidelines.

## Patients

### Inclusion criteria

Positive SARS-CoV-2 patients were diagnosed via qRT-PCR. Only positive patients with typical symptoms who met the following criteria were enrolled: 18–65 years old; less than 24 h after illness onset; positive for SARS-CoV-2 with typical symptoms, including fever, cough, sore throat, and fatigue and signed written consent.

### Exclusion criteria

Patients who had the following diseases were removed: treated with any known anti-viral drugs or convalescent plasma before enrollment; had severe infectious diseases, immunodeficiency, malignant tumors, organ or bone marrow transplants, AIDS, or other immunosuppressive diseases; had serious cardiovascular and cerebrovascular diseases, chronic lung diseases, poorly controlled diabetes, or other severe diseases; had known allergies to the ingredients of the investigational drugs; had any gastrointestinal diseases; and were pregnant or breastfeeding.

### Withdrawal (dropout) criteria

Patients who have the following conditions will be withdrawn. Patients with serious complications, special physiological changes, serious adverse events, or severe allergic reactions; patients who seriously violated the inclusion or exclusion criteria after randomization; those who had no visit records; patients with poor compliance and who were deemed unsuitable for continued participation by the investigator; and patients who were unwilling to continue trial participation.

### Randomization

All recruited patients were randomly divided into FFYH or LHQW groups according to random numbers, which the contract research organization generated via the block randomization function of SAS version 9.4 (SAS Institute, Cary, NY). During the trial, all patients were prohibited from changing the drug number, and all trial drugs were from the same lot number. To ensure uniformity in allocation, random numbers were placed in opaque sealed envelopes and then uniformly numbered and distributed to the clinicians. Three professional statisticians analyzed all the data.

### Intervention

Two hundred recruited patients who met all the inclusion criteria were assigned randomly at a 1:1 ratio to receive either FFYH (oral, 1 bag (15 g/bag)/dose, thrice daily, produced by Hefei Yifan Pharmaceutical Co., Ltd.) or LHQW granules (oral, 1 bag (6 g/bag)/dose, thrice daily, manufactured by Beijing Yiling Pharmaceutical Co., Ltd.) for 3–7 consecutive d. The chemical composition and HPLC profiles of FFYH were confirmed in our previous work (Zhang et al., 2021).

## Clinical management and outcomes

All patients underwent daily examinations, including clinical symptoms, blood pressure, heart rate, respiration rate, and body temperature. Drug compliance and adverse events during the treatment periods were recorded. The primary clinical endpoints included recovery time and improvement in clinical symptoms. The secondary endpoints were the negative conversion time of SARS-CoV-2 RNA and the percentage of SARS-CoV-2-negative patients, changes in hematology parameters, changes in serum cytokines, and the percentage of severe or critical cases.

### Efficacy judgment criteria

Efficacy judgment criteria include the following: Time for fever relief, the time required for the body temperature of the subject to return to normal (<37.3°C) for more than 48 h after the first administration. Time for respiratory symptom resolution, the time required for symptoms to resolve for more than 24 h, such as cough, dry throat, sore throat, nasal obstruction, and rhinorrhea; time to clinical symptom resolution, the time required for symptoms to resolve for more than 24 h, such as fever, fatigue, cough, dried pharynx, sore throat, nasal congestion, runny nose, taste disorder, olfactory disorder, and other symptoms; and negative conversion of the RT-PCR test for SARS-CoV-2; the RT-PCR test for SARS-CoV-2 is negative from the first administration. Incidence of complications, common cases or severe cases, and critical cases: incidence of complications = number of subjects with complications in each treatment group/total number of subjects in this treatment group. For the definitions of common or severe and critical COVID-19 cases, refer to the diagnostic criteria in the *Diagnosis and Treatment Protocol for COVID-19 Patients (Tentative 9th Edition)*.

### Statistical analysis

To avoid the influence of subjective factors on the analysis of the experimental data, all the clinical data were analyzed independently by three professional statisticians from Beijing Yurong Medical Data Technology Co., Ltd., via SAS version 9.4. All experimental results are expressed as the mean ± standard deviation (SD). For multiple groups, significant differences were evaluated by one-way analysis of variance (ANOVA) or Student’s t-test, and *P <* 0.05 was considered statistically significant.

## Results

### Anti-viral effect of FFYH on SARS-CoV-2

A virus titer assay indicated that FFYH (1,000 μg/mL, 500 μg/mL and 250 μg/mL) inhibited the release of newborn SARS-CoV-2 in a dose-dependent manner (Figure 1A). Moreover, FFYH dose-dependently reduced the RNA (Figure 1B) and protein levels of SARS-CoV-2 (Figures 1C, D). A time-of-drug-addition assay indicated that FFYH pretreatment (1,000 μg/mL and 500 μg/mL) has an obvious inhibitory effect on the replication of SARS-CoV-2, whereas FFYH coincubation or posttreatment has a significant inhibitory effect, suggesting that FFYH mainly targets the binding and replication stage of the SARS-CoV-2 life cycle (Figures 1E–I). Moreover, the inhibitory effect of FFYH posttreatment was greater than that of FFYH coincubation, indicating that FFYH mainly targeted the replication stage of SARS-CoV-2. Immunofluorescence assays further confirmed the anti-SARS-CoV-2 effect of FFYH (Figures 1J, K).

### Anti-viral mechanism of FFYH against SARS-CoV-2

**FIGURE 2:**
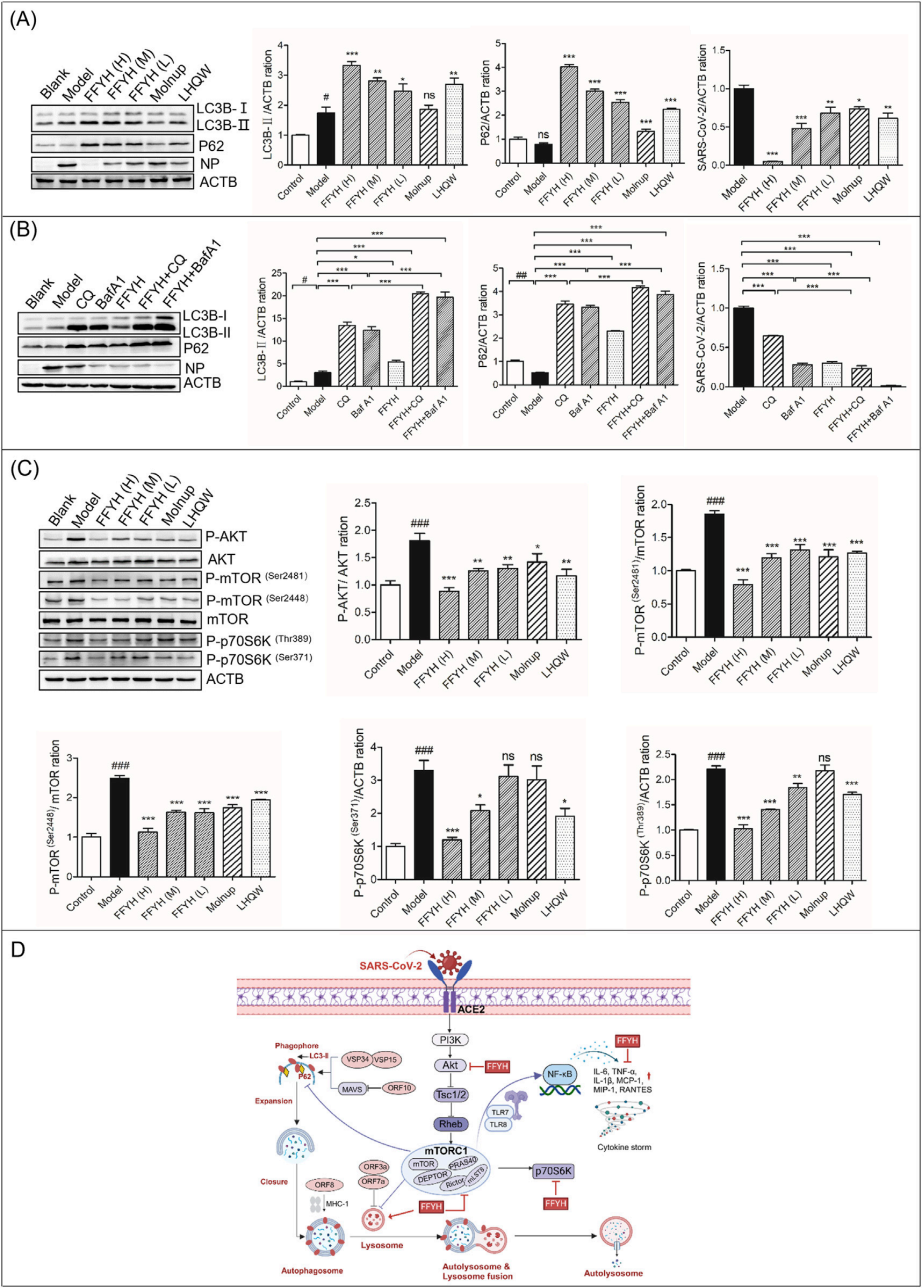
Anti-viral mechanism of action of FFYH against SARS-CoV-2. **(A)** FFYH blocked autophagy to inhibit the replication of SARS-CoV-2. **(B)** Blocking the formation of autophagolysosomes synergistically enhances the anti-viral effect of FFYH against SARS-CoV-2. **(C)** Inhibiting the PI3K/AKT/mTOR signaling pathway may be the primary mechanism by which FFYH blocks SARS-CoV-2-triggered autophagy. **(D)** Potential model of the effects of FFYH against SARS-CoV-2 infection. ^ns^ *P*>0.05, ^#^ *P* < 0.05, and ^##^ *P* < 0.01, compared with the normal control group; ^ns^ *P*>0.05, ^*^ *P* < 0.05, ^**^ *P* < 0.01, and ^***^ *P* < 0.001, compared with the virus-infected group.

Autophagy is involved in the clearance and recycling of intracellular materials and the elimination of intracellular pathogens. Many studies have reported that SARS-CoV-2 can hijack autophagy to promote replication via multiple mechanisms, suggesting that blocking autophagy may be a potential target for treating COVID-19 (Gassen et al., 2021). Therefore, we first investigated whether FFYH affects SARS-CoV-2-triggered autophagy. Our results indicated that SARS-CoV-2 infection markedly promoted LC3B-II accumulation but induced P62 degradation (Figure 2A), suggesting that SARS-CoV-2 triggered autophagy. However, FFYH dose-dependently increased LC3B-II and P62 protein accumulation but inhibited SARS-CoV-2 replication (Figure 2A), suggesting that FFYH blocked SARS-CoV-2-triggered autophagy. To further confirm these results, we used CQ and BafA1 pretreatment to investigate the influence of FFYH against SARS-CoV-2. Compared with CQ or BafA1 treatment alone, FFYH combined with BafA1 synergistically enhanced the anti-SARS-CoV-2 effect of FFYH (Figure 2B), suggesting that blocking SARS-CoV-2-triggered autophagy may be the primary mechanism by which FFYH affects SARS-CoV-2.

The PI3K/Akt/mTOR pathway is considered a possible target for the treatment of COVID-19. Therefore, we first investigated whether FFYH affects the PI3K/AKT/mTOR pathway. Our results indicated that SARS-CoV-2 infection markedly activated this pathway (Figure 2C). However, FFYH inhibited the activation of this pathway in a dose-dependent manner (Figure 2C). Therefore, we speculated that inhibiting the PI3K/AKT/mTOR signaling pathway may be the primary mechanism by which FFYH blocks SARS-CoV-2-triggered autophagy (Figure 2D).

## Patients’ baseline characteristics

**FIGURE 3:**
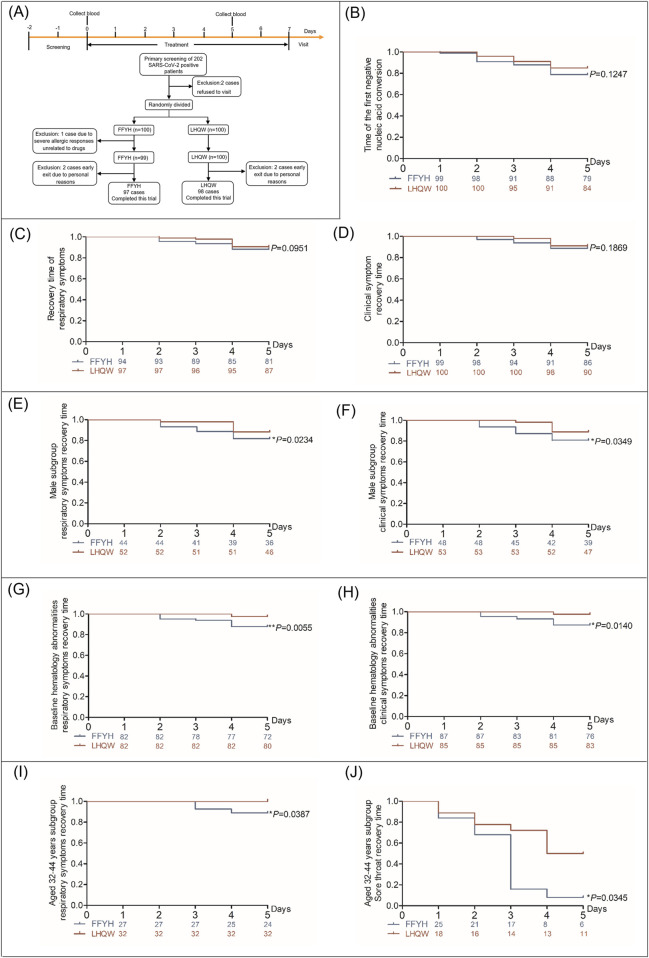
Clinical efficacy of FFYH in mild COVID-19 patients. **(A)** Flowchart of screening, randomization, and treatment of mild COVID-19 patients. **(B)** Time to first nucleic acid conversion in COVID-19 patients treated with FFYH. **(C)** Recovery time of respiratory symptoms in COVID-19 patients treated with FFYH. **(D)** Recovery time of clinical symptoms in COVID-19 patients treated with FFYH. **(E)** Recovery time of respiratory symptoms in male COVID-19 patients treated with FFYH. **(F)** Recovery time of clinical symptoms in male COVID-19 patients treated with FFYH. **(G)** Recovery time of respiratory symptoms in COVID-19 patients with baseline hematology abnormalities after treatment with FFYH. **(H)** Recovery time of clinical symptoms in COVID-19 patients with baseline hematology abnormalities after treatment with FFYH. **(I)** Recovery time of respiratory symptoms in COVID-19 patients aged 33–42 years treated with FFYH. **(J)** Recovery time of sore throat symptoms in COVID-19 patients aged 33–42 years treated with FFYH.

A total of 202 mildly symptomatic COVID-19 patients were recruited, and only two patients were excluded. Two hundred patients were successfully enrolled from 12 December 2022 to 26 December 2022 and were randomly assigned to the FFYH or LHQW treatment group. During the trial, one patient was withdrawn from the trial in advance due to other severe allergic reactions. Four patients were withdrawn from the trial for personal reasons during the observation period. A total of 195 patients completed this trial (Figure 3A).

The general information on the patients is summarized in Table 1. A total of 198 patients received COVID-19 vaccination. All patients had no history of alcohol abuse, drug abuse, mental illness, or surgery. Two patients had accompanying medical histories. No patients had participated in other drug clinical trials before enrollment. A total of 172 patients had abnormal hematology parameters at baseline. No significant differences in sex ratio, age, height, weight, past medical history, BMI, pulse, respiration, diastolic pressure, body temperature, or initial symptoms were detected between the FFYH and LHQW groups at baseline. However, significant differences in baseline systolic pressure and sore throat were observed between the FFYH and the LHQW groups (Table 1).

**TABLE 1:** Clinical characteristics of patients with mild COVID-19.

Term	FFYH (N = 99)	LHQW (N = 100)	*p*-value
Male (%)	48 (48.5)	53 (53.0)	0.5241
Age (M±SD)	39.1 ± 11.17	37.6 ± 11.0	0.3469
Height (cm, M±SD)	166.9 ± 8.0	168.1 ± 8.0	0.2819
Weight (kg, M±SD)	65.5 ± 11.8	68.2 ± 13.2	0.1259
Han nationality^a^	99 ± 0.0	100 ± 0.0	-
BMI (kg/m^2^, M±SD)	23.4 ± 3.2	24.03 ± 3.7	0.1937
Pulse (times/min, M±SD)	86.6 ± 10.9	85.4 ± 10.9	0.4256
Respiratory (times/min, M±SD)	15.8 ± 1.6	16.0 ± 1.5	0.4083
Systolic pressure (mmHg, M±SD)	123.5 ± 12.0	127.6 ± 12.3	0.0184^*^
Diastolic pressure (mmHg, M±SD)	78.5 ± 7.3	79.8 ± 8.1	0.2381
Temperature (°C, M±SD)	37.3 ± 0.7	37.2 ± 0.8	0.5901
Past medical history (%)			
Asthma	0 (0.0)	1 (0.5)	-
Hypertension	0 (0.0)	1 (0.5)	-
Initial symptoms (%)			
Fever	47 (47.5)	37 (37.0)	0.1347
Fatigue	70 (70.7)	70 (70.0)	0.9130
Cough	80 (80.8)	84 (84.0)	0.5543
Dried pharynx	81 (81.8)	74 (74.0)	0.1839
Sore throat	66 (66.7)	52 (52.0)	0.0352^*^
Nasal congestion	43 (43.4)	53 (53.0)	0.1769
Runny nose	41 (41.4)	43 (43.0)	0.8208
Taste disorder	30 (30.3)	19 (19.0)	0.0642
Olfactory disorder	6 (6.1)	6 (6.0)	0.9857

^*^ *P* < 0.05, FFYH, vs. LHQW.

^a^ As reported by the patient.

### Efficacy analysis

No patients progressed to severe disease, and no statistically significant difference in the nucleic acid conversion to negative time or the recovery time of clinical symptoms was observed between FFYH and LHQW treatment (Figures 3B–D). However, sex subgroup analysis revealed that the recovery time of clinical symptoms and respiratory symptoms in the FFYH-treated male subgroup was shorter than that in the LHQW-treated male subgroup (Figures 3E, F, *p* < 0.05), whereas no statistically significant differences were detected between the two female subgroups (Table 2). In addition, the recovery times of patients with clinical symptoms (*P* < 0.05), respiratory symptoms (*P* < 0.01), and cough (*P* < 0.05) in the FFYH-treated group with baseline hematology abnormalities were significantly shorter than those in the LHQW-treated group (Figures 3G, H; Table 2). Age subgroup analysis revealed that the recovery time of respiratory symptoms and sore throat of 32–42-year-old patients treated with FFYH was significantly shorter than that of patients treated with LHQW (Figure 3I, J, *P* < 0.05). Collectively, these results indicate that FFYH may be more effective than LHQW in improving the clinical symptoms of male patients, patients with abnormal hematology parameters, and those aged 32–42 years. Improvements in the clinical symptoms of the different groups are shown in Supplementary Table 1-7 1.

**TABLE 2:** Improvement in clinical symptoms in different subgroups of COVID-19 patients treated with FFYH or LHQW.

Term	FFYH	LHQW	*p*-value
Male subgroup	N = 48	N = 53	
Clinical symptom recovery (%)	26 (54.2)	18 (34.0)	0.0349*
Time to clinical symptom recovery (d, M±SD)	5.3 ± 1.85	5.8 ± 1.47	
Respiratory symptom recovery (%)	25 (56.8)	18 (34.6)	0.0234*
Time to respiratory symptom recovery (d, M±SD)	5.3 ± 1.79	5.8 ± 1.59	
Female subgroup	N = 51	N = 47	
Fatigue (%)	25 (64.1)	32 (86.5)	0.0278*
Time to fatigue recovery (d, M±SD)	3.5 ± 1.48	3.1 ± 1.95	
Baseline hematology abnormality subgroup	N = 87	N = 85	
Clinical symptom recovery (%)	38 (43.7)	24 (28.2)	0.0140*
Time to clinical symptom recovery (d, M±SD)	5.7 ± 1.63	6.3 ± 1.09	
Respiratory symptom recovery (%)	38 (46.3)	23 (28.0)	0.0055**
Time to respiratory symptom recovery (d, M±SD)	5.7 ± 1.58	6.4 ± 1.08	
Cough (%)	34 (50.0)	25 (35.2)	0.0352*
Time to cough recovery (d, M±SD)	4.6 ± 2.07	4.8 ± 2.14	
33∼42-year-old subgroup	N = 29	N = 33	
Respiratory symptom recovery (%)	11 (40.7)	5 (15.6)	0.0387*
Time to respiratory symptom recovery (d, M±SD)	5.6 ± 1.63	6.0 ± 1.00	
Sore throat (%)	21 (91.3)	13 (76.5)	0.0345*
Time to sore throat recovery (d, M±SD)	3.1 ± 1.65	4.2 ± 2.15	

^*^ *P* <0.05,

^**^ *P* <0.001.

### Improvements in hematology parameters

FFYH or LHQW treatment markedly improved patients’ hematology parameters by increasing the lymphocyte (Lymph) and platelet (PLT) counts and reducing the neutrophil (NE), monocyte (Mon), neutrophil/lymphocyte ratio (NLR), monocyte/lymphocyte ratio (MLR), platelet count/lymphocyte ratio (PLR), and neutrophil×platelet count/lymphocyte ratio (SII) (Table 3). Sex subgroup analysis revealed that the HGB and HCT levels in the LHQW-treated female subgroup were significantly lower (*P* < 0.05 or 0.001) than those in the FFYH-treated female subgroup, whereas no statistically significant differences were detected between the two male subgroups (Table 3).

**TABLE 3:** Improvement in hematology parameters in COVID-19 patients treated with FFYH or LHQW.

Term	FFYH(S) (N = 97)	FFYH(E) (N = 97)	*p*-Value	LHQW(S) (N = 99)	LHQW(E) (N = 99)	*p*-value
Total						
HGB (g/L)	146.1 ± 16.5	158.3 ± 12.3	ns	148.7 ± 14.6	147.9 ± 17.0	ns
HCT%	43.0 ± 4.7	46.9 ± 3.8	ns	43.9 ± 4.2	43.8 ± 4.8	ns
RBC (×1,012/L)	4.8 ± 0.6	5.2 ± 0.5	ns	4.9 ± 0.5	4.9 ± 0.5	ns
WBC (×109/L)	5.7 ± 1.8	6.0 ± 1.4	ns	5.7 ± 1.7	5.7 ± 1.7	ns
PLT (×109/L)	201.3 ± 52.3	223.5 ± 56.0↑	<0.001***	200.5 ± 52.4	231.4 ± 68.3↑	<0.001***
NE (×109/L)	3.7 ± 1.5	3.2 ± 1.1↓	<0.01**	3.9 ± 1.6	3.1 ± 1.3↓	<0.01**
LymPh (×109/L)	1.2 ± 0.5	2.2 ± 0.6↑	<0.001***	1.2 ± 0.5	2.1 ± 0.7↑	<0.001***
Mon (×109/L)	0.6 ± 0.3	0.4 ± 0.1↓	<0.001***	0.6 ± 0.2	0.3 ± 0.1↓	<0.001***
NLRb	4.0 ± 3.0	1.51 ± 0.68↓	<0.001***	4.1 ± 2.9	1.61 ± 0.78↓	<0.001***
MLRc	0.6 ± 0.4	0.16 ± 0.07↓	<0.001***	0.6 ± 0.3	0.2 ± 0.1↓	<0.001***
PLRd	205.8 ± 134.0	115.0 ± 38.1↓	<0.001***	199.4 ± 106.1	120.4 ± 50.5↓	<0.001***
SⅡe	803.0 ± 667.0	355.4 ± 201.6↓	<0.001***	834.2 ± 692.6	383.8 ± 234.6↓	<0.001***
Male subgroup	N = 47	N = 47		N = 53	N = 53	
HGB (g/L)	158.4 ± 11.1	158.3 ± 12.3	ns	158.2 ± 9.8	159.7 ± 11.0	ns
HCT%	46.6 ± 3.3	46.9 ± 3.8	<0.001***	46.7 ± 3.0	47.1 ± 3.4	<0.001***
RBC (×1,012/L)	5.1 ± 0.5	5.2 ± 0.5	ns	5.1 ± 0.4	5.2 ± 0.4	ns
WBC (×109/L)	6.2 ± 1.6	6.0 ± 1.4	ns	5.9 ± 1.8	5.9 ± 1.7	ns
PLT (×109/L)	191.9 ± 39.0	223.5 ± 56.0↑	<0.001***	194.3 ± 42.6	219.8 ± 51.6↑	<0.001***
NE (×109/L)	4.0 ± 1.5	3.2 ± 1.1↓	<0.001***	3.9 ± 1.6	3.2 ± 1.3↓	<0.001***
LymPh (×109/L)	1.4 ± 0.5	2.2 ± 0.6↑	<0.001***	1.3 ± 0.5	2.2 ± 0.7↑	<0.001***
Mon (×109/L)	0.7 ± 0.3	0.4 ± 0.1↓	<0.001***	0.6 ± 0.3	0.4 ± 0.1↓	<0.001***
NLRb	3.5 ± 2.7	1.5 ± 0.7↓	<0.001***	3.6 ± 2.6	1.6 ± 0.8↓	<0.001***
MLRc	0.6 ± 0.3	0.1 ± 0.1↓	<0.001***	0.5 ± 0.3	0.1 ± 0.1↓	<0.001***
PLRd	162.9 ± 97.8	107.0 ± 37.9↓	<0.001***	172.8 ± 91.1	108.4 ± 39.0↓	<0.001***
SⅡe	690.4 ± 557.3	354.3 ± 199.5↓	<0.001***	739.9 ± 672.7	349.3 ± 175.6↓	<0.001***
Female subgroup	N = 50	N = 50		N = 46	N = 46	
HGB (g/L)	134.6 ± 11.8	133.7 ± 11.6	ns	137.9 ± 11.3	134.3 ± 11.7↓	<0.001***
HCT%	39.7 ± 3.1	39.7 ± 3.1	ns	40.7 ± 3.1	40.0 ± 3.2↓	<0.05*
RBC (×1,012/L)	4.4 ± 0.4	4.4 ± 0.4	ns	4.5 ± 0.4	4.5 ± 0.4	ns
WBC (×109/L)	5.1 ± 1.8	5.3 ± 1.8	ns	5.5 ± 1.7	5.4 ± 1.7	ns
PLT (×109/L)	210.1 ± 61.4	238.4 ± 71.2↑	<0.001***	207.7 ± 61.6	244.8 ± 82.2↑	<0.001***
NE (×109/L)	3.5 ± 1.6	2.9 ± 1.3↓	<0.01**	3.8 ± 1.7	3.0 ± 1.3↓	<0.01**
LymPh (×109/L)	1.0 ± 0.5	2.0 ± 0.6↑	<0.001***	1.1 ± 0.5	2.0 ± 0.6↑	<0.001***
Mon (×109/L)	0.5 ± 0.2	0.3 ± 0.1↓	<0.001***	0.5 ± 0.2	0.3 ± 0.1↓	<0.001***
NLR^b^	4.4 ± 3.3	1.5 ± 0.7↓	<0.001***	4.6 ± 3.0	1.6 ± 0.8↓	<0.001***
MLR^c^	0.6 ± 0.36	0.16 ± 0.07↓	<0.001***	0.6 ± 0.4	0.16 ± 0.07↓	<0.001***
Female subgroup	N = 50	N = 50		N = 46	N = 46	
PLR^d^	246.2 ± 150.9	124.4 ± 36.8↓	<0.001^***^	230.1 ± 114.7	134.2 ± 58.5↓	<0.001^***^
SⅡ^e^	908.9 ± 746.0	356.5 ± 205.6↓	<0.001^***^	842.9 ± 706.5	423.5 ± 285.0↓	<0.001^***^

^b^ Neutrophil / lymphocyte ratio.

^c^ Monocyte/lymphocyte ratio.

^d^ Platelet count/lymphocyte ratio.

^e^ (Neutrophil × platelet count)/lymphocyte.

^**^ *P* <0.01,

^***^ *P* <0.001.

### Improvement in excessive inflammatory cytokines

Compared with those in healthy individuals, the levels of IFN-γ, IL-12 (p70), IL-13, IL-1β, IL-2, IL-4, IL-5, IL-6, GM-CSF, IL-17A, IL-27, IFN-α, IL-15, IL-1RA, IL-7, eotaxin, GRO-α, IP-10, MCP-1, MIP-1α, MIP-1β, SDF-1α, and RANTES in mild COVID-19 patients were significantly greater. Compared with the baseline levels, after treatment with FFYH or LHQW, the serum levels of inflammatory cytokines significantly improved, as indicated by significant increases in the levels of IFN-γ, IL-12p70, IL-13, IL-1β, IL-2, IL-4, IL-5, IL-6, IL-15, IL-17A, and IL-27 (*P* < 0.05 or 0.01) but markedly decreased levels of GM-CSF, IFN-α, IL-1RA, IL-7, eotaxin, GRO-α, IP-10, MCP-1, MIP-1α, MIP-1β, SDF-1α, and RANTES (*P* < 0.05 or 0.001) (Table 4). However, significant differences in elevated IL-12p70, IL-13, IL-4, and IL-6 levels were observed between the FFYH- and LHQW-treated groups, suggesting that the FFYH-treated group had greater improvements in the levels of IL-12p70, IL-13, IL-4, and IL-6 than the LHQW-treated group. Notably, the IL-6 levels in the LHQW-treated group were significantly elevated compared with the baseline levels in this group (*P* < 0.001), whereas the IL-6 levels in the FFYH-treated group slightly increased, suggesting that FFYH may be superior to LHQW in controlling the IL-6 levels (*P* < 0.05) (Table 4). Furthermore, FFYH treatment had a more significant inhibitory effect on GM-CSF than LHQW treatment (Table 4), but LHQW treatment had a greater inhibitory effect on SDF-1α than FFYH.

**TABLE 4:** Improvement in cytokine levels in COVID-19 patients treated with FFYH or LHQW.

Term	FFYH(S) (N = 95)	FFYH(E) (N = 95)	*p*-value	LHQW(S) (N = 96)	LHQW(E) (N = 96)	*p*-value
Total						
IL-12P70	3.7 ± 1.1	4.0 ± 1.0↑	<0.01**	3.8 ± 1.1	4.4 ± 1.1↑	<0.001***
IL-13	15.7 ± 9.8	18.6 ± 9.0↑	<0.01**	17.1 ± 10.9	22.1 ± 11.0↑	<0.001***
IL-4	5.1 ± 5.5	7.0 ± 5.8↑	<0.05*	5.9 ± 5.6	8.2 ± 5.5↑	<0.001***
IL-6	17.2 ± 19.0	18.6 ± 17.5	ns	22.2 ± 37.8	28.2 ± 28.5↑	<0.001***
IL-1β	6.7 ± 4.2	8.6 ± 4.4↑	<0.001***	7.0 ± 3.8	9.5 ± 4.3↑	<0.001***
IL-2	18.6 ± 10.3	24.0 ± 10.2↑	<0.001***	20.5 ± 11.1	25.23 ± 10.4↑	<0.001***
IL-5	21.4 ± 12.4	27.2 ± 11.3↑	<0.001***	22.2 ± 11.6	28.5 ± 12.1↑	<0.001***
IFN-γ	22.8 ± 15.2	30.3 ± 14.4↑	<0.001***	25.7 ± 15.4	33.4 ± 13.9↑	<0.001***
IL-17A	17.6 ± 11.5	21.6 ± 9.3↑	<0.001***	18.3 ± 10.4	24.9 ± 12.3↑	<0.001***
IL-27	43.3 ± 32.8	56.4 ± 32.8↑	<0.001***	45.8 ± 37.2	67.8 ± 45.4↑	<0.001***
IL-15	10.7 ± 6.5	13.5 ± 5.7↑	<0.001***	11.5 ± 5.9	14.6 ± 6.2↑	<0.001***
GM-CSF	22.8 ± 11.4	18.2 ± 8.9↓	<0.001***	24.7 ± 14.7	21.5 ± 13.4↓	<0.05*
IFN-α	4.7 ± 8.1	0.5 ± 0.3↓	<0.001***	5.1 ± 10.9	0.6 ± 0.3↓	<0.001***
IL-1RA	1,305.0 ± 1,417.0	285.6 ± 177.4↓	<0.001***	1,185.7 ± 1,387.0	316.5 ± 246.2↓	<0.001***
IL-7	3.7 ± 1.6	2.8 ± 1.1↓	<0.001***	4.0 ± 2.5	2.9 ± 1.1↓	<0.001***
Eotaxin	20.5 ± 7.6	15.1 ± 5.0↓	<0.001***	20.8 ± 10.7	15.2 ± 6.6↓	<0.001***
GRO-α	3.7 ± 6.0	0.8 ± 1.7↓	<0.001***	3.6 ± 4.0	0.9 ± 1.7↓	<0.001***
IP-10	171.3 ± 118.0	33.5 ± 16.9↓	<0.001***	160.67 ± 116.54	34.2 ± 20.3↓	<0.001***
MCP-1	126.0 ± 106.0	52.3 ± 23.9↓	<0.001***	131.7 ± 117.98	59.2 ± 55.0↓	<0.001***
MIP-1α	1.3 ± 1.4	0.4 ± 0.9↓	<0.001***	1.5 ± 2.8	0.6 ± 1.5↓	<0.001***
MIP-1β	62.3 ± 47.4	37.2 ± 26.0↓	<0.001***	59.2 ± 50.1	37.7 ± 30.8↓	<0.001***
SDF-1α	661.9 ± 1,415.0	572.2 ± 1884.1	ns	471.6 ± 517.0	357.3 ± 239.1↓	<0.01**
RANTES	134.3 ± 32.6	121.5 ± 31.9↓	<0.001***	133.0 ± 30.9	118.7 ± 35.6↓	<0.001***
Male subgroup	N = 49	N = 49	*p*-value	N = 51	N = 51	*p*-value
IL-1β	6.3 ± 4.1	8.6 ± 4.3↑	<0.001***	6.9 ± 3.6	9.2 ± 4.3↑	<0.01**
IL-6	15.5 ± 18.5	16.4 ± 19.1	ns	22.3 ± 37.0	29.6 ± 31.3↑	<0.05*
TNF-α	8.7 ± 4.0	9.9 ± 4.2	ns	9.8 ± 4.5	11.3 ± 4.3↑	<0.05*
IL-1α	0.3 ± 0.4	0.4 ± 0.5↑	<0.01**	0.3 ± 0.2	0.4 ± 0.2↑	<0.05*
IL-27	36.4 ± 30.2	53.0 ± 31.3↑	<0.01**	49.7 ± 44.6	70.6 ± 51.9↑	<0.001***
IL-31	0.3 ± 0.7	0.3 ± 0.7	ns	1.5 ± 4.7	0.6 ± 2.2↓	<0.05*
GM-CSF	24.2 ± 12.2	19.4 ± 8.4↓	<0.001***	26.6 ± 17.4	25.2 ± 15.7	ns
MIP-1β	66.2 ± 64.3	39.6 ± 35.6↓	<0.001***	65.8 ± 65.9	42.8 ± 40.6↓	<0.001***
IL-7	3.4 ± 1.4	2.6 ± 1.0↓	<0.001***	3.8 ± 2.4	2.9 ± 1.1↓	<0.01**
SDF-1α	839.2 ± 1930.2	764.2 ± 2,689.0	ns	525.9 ± 683.3	364.5 ± 274.1↓	<0.05*
RANTES	135.2 ± 29.9	118.7 ± 29.3↓	<0.001***	135.3 ± 31.5	119.6 ± 27.4↓	<0.01**
**Female subgroup**	N = 46	N = 46	*p*-value	N = 45	N = 45	*p*-value
IFN-γ	24.7 ± 14.9	31.7 ± 15.1**↑**	<0.05^*^	26.5 ± 15.6	35.0 ± 13.7**↑**	<0.001^***^
IL-12P70	3.8 ± 1.2	4.1 ± 1.0**↑**	<0.05^*^	3.7 ± 1.3	4.5 ± 1.3**↑**	<0.01^**^
IL-13	16.2 ± 9.4	19.1 ± 8.4	ns	16.1 ± 10.7	20.9 ± 10.4**↑**	<0.01^**^
IL-1β	7.2 ± 4.3	8.6 ± 4.5**↑**	<0.05^*^	7.1 ± 4.1	9.8 ± 4.3**↑**	<0.001^***^
IL-2	19.3 ± 9.7	24.6 ± 10.1**↑**	<0.01^**^	21.1 ± 12.3	25.6 ± 9.8**↑**	<0.05^*^
IL-4	6.0 ± 6.0	7.5 ± 5.7	ns	5.6 ± 4.9	8.2 ± 5.4**↑**	<0.01^**^
IL-17A	18.5 ± 11.2	21.8 ± 9.5**↑**	<0.05^*^	17.5 ± 9.4	24.8 ± 12.3**↑**	<0.001^***^
IL-27	50.0 ± 34.0	59.6 ± 34.2	ns	41.4 ± 26.4	64.6 ± 36.9**↑**	<0.001^***^
GM-CSF	21.5 ± 10.5	17.0 ± 9.3↓	<0.001^***^	22.7 ± 10.8	17.3 ± 8.4↓	<0.01^**^
IFN-α	5.8 ± 9.5	0.5 ± 0.3↓	<0.001^***^	6.1 ± 13.4	0.6 ± 0.3↓	<0.01^**^
SDF-1α	495.5 ± 610.3	392.0 ± 323.2↓	<0.05^*^	410.1 ± 197.8	349.1 ± 194.9	ns

^*^ *P*<0.05,

^**^ *P*<0.01,

^***^ *P* < 0.001, FFYH, vs. LHQW.

Analysis of sex differences revealed significant differences in the expression levels of IL-1β, IL-6, TNF-α, IL-1α, IL-27, IL-31, GM-CSF, IL-7, SDF-1α, and RANTES between male patients treated with FFYH and those treated with LHQW. Compared with their baseline levels, the IL-6, TNF-α, IL-31, and SDF-1α levels in the FFYH-treated male subgroup were slightly elevated or decreased (*P* > 0.05), whereas the expression of these cytokines in the corresponding LHQW-treated group was markedly increased or decreased (*P* < 0.05) (Table 4). Furthermore, the GM-CSF, IL-7, and RANTES levels in the FFYH-treated male subgroup were markedly lower than their baseline levels were (*P* < 0.001), indicating a stronger inhibitory effect than that in the corresponding LHQW-treated group (Table 4). Moreover, significant sex differences were also observed between the FFYH-treated and LHQW-treated female subgroups. Compared with their baseline levels, the expression levels of IL-13, IL-4, and IL-27 in the FFYH-treated female group were slightly increased (*P* > 0.05), whereas the levels of these cytokines in the corresponding LHQW-treated subgroup were significantly increased (*P* < 0.05) (Table 4). Moreover, the levels of IFN-γ, IL12p70, IL-1β, and IL-17A in the LHQW-treated female subgroup were greater than those in the FFYH-treated female subgroup and their baseline levels. In addition, FFYH had a stronger inhibitory effect on GM-CSF, SDF-1α, and IFN-α expression in female patients than LHQW (Table 4). These results indicate that FFYH treatment may be effective in controlling excessive inflammatory responses in COVID-19 patients.

## Discussion

TCM has been widely used in the clinical prevention and treatment of respiratory tract infections (Yu et al., 2014; Lyu et al., 2021; Wang et al., 2014). Many studies have indicated that TCM is effective for treating infectious diseases such as influenza (Li et al., 2016), severe acute respiratory syndrome (SARS) (Zhang et al., 2004), and respiratory syncytial virus (RSV) infections (Lin et al., 2016). In addition, clinical practices in China have indicated that TCM plays a critical role in the treatment of COVID-19 (Lyu et al., 2021; Xing and Liu, 2021; Zhang et al., 2020a). FFYH derived from “Yin-Qiao-San” has also been recommended for the prevention and treatment of COVID-19 in China. Our previous work demonstrated that FFYH could effectively inhibit the replication of coronaviruses (Zheng et al., 2022). However, the clinical efficacy of FFYH in patients with COVID-19 remains to be confirmed. Here, our results indicated that *in vitro* FFYH can dose-dependently inhibit the replication of SARS-CoV-2 by primarily targeting the stage of virus replication. Mechanistically, our results revealed that blocking SARS-CoV-2-triggered autophagy is the primary mechanism by which FFYH protects against SARS-CoV-2 infection via the inhibition of the PI3K/Akt/mTOR signaling pathway.

Moreover, the clinical efficacy of FFYH for the treatment of COVID-19 has been confirmed. Our results demonstrated that FFYH significantly improved the clinical symptoms of mild COVID-19 patients by shortening the recovery time of clinical symptoms, promoting SARS-CoV-2 RNA conversion negativity, improving hematology parameters, and controlling excessive cytokine storms. Moreover, our results indicated that, compared with the LHQW positive control, FFYH had a more significant improvement effect on clinical symptoms and respiratory symptoms in male patients, patients with abnormal hematology parameters, and those aged 32–42 years. Epidemiological and clinical studies have indicated that SARS-CoV-2 infection results in more severe outcomes and higher mortality rates in men than in women (Chen et al., 2020). Therefore, we speculate that FFYH may be more suitable for improving the clinical symptoms of male patients with COVID-19.

Hematological parameters play crucial roles in assessing the progression of COVID-19 patients from mild to severe disease or in evaluating the effectiveness of pharmacological treatments (Djakpo et al., 2020). Our study results indicate that the lymphocyte, PLT, NE, Mon, NLR, MLR, PLR, and SII levels significantly decrease after treatment with FFYH and LHQW. This finding is consistent with earlier studies. However, the levels of HGB and HCT in the LHQW-treated female subgroup significantly decreased. HGB is reportedly an effective hematological parameter for distinguishing severe from non-severe COVID-19 (Zhang et al., 2020b). Moreover, HGB and HCT levels in female patients may be more susceptible to the effects of viral infections (Djakpo et al., 2020). Furthermore, a lower HGB level is associated with a more severe disease course and a higher mortality rate (Algassim et al., 2021). Therefore, FFYH may be more suitable for female COVID-19 patients. However, the exact mechanism by which FFYH improves HGB and HCT levels still requires further investigation.

Cytokine storms are a leading cause of ARDS and multiple organ failure (Kim et al., 2021; Yang et al., 2021). Studies have indicated that GM-CSF is a key cytokine involved in the inflammatory COVID-19 response and may indirectly lead to ARDS by inhibiting neutrophil apoptosis (Dharra et al., 2023). Moreover, the expression of proinflammatory cytokines such as IL-1RA, IFN-α, IL-7, IP-10, MIP-1α, and MIP-1β is strongly correlated with the severity of COVID-19. These cytokines are considered biomarkers of the clinical severity of patients (Yang et al., 2020; Fernandes and Barata, 2023; Krämer et al., 2021). Therefore, timely control of excessive cytokine storms may be an effective approach for preventing COVID-19 exacerbation (Kim et al., 2021). Therefore, many new targeted anti-inflammatory therapies have been investigated for suppressing excessive immune responses (Zhu et al., 2023; Michot et al., 2020). However, the immunosuppressive effect of anti-inflammatory therapy may paradoxically impede viral clearance and increase the risk of secondary bacterial infection. Additionally, steroids (methylprednisolone and dexamethasone) are also primary treatments for COVID-19, but studies have shown that methylprednisolone and dexamethasone carry an increased risk of thrombosis (Jiang et al., 2022). Here, our results revealed that SARS-CoV-2 infection resulted in excessive cytokine storms in all vaccinated COVID-19 patients. However, FFYH effectively balanced the cytokine levels by slightly upregulating the expression of IFN-γ, IL-1β, IL-2, IL-5, IL-15, IL-17A, and IL-27 and by markedly inhibiting GRO-α, IL-7, IL-1RA, IFN-α, GM-CSF, eotaxin, IP-10, MCP-1, MIP-1α, MIP-1β, RANTES, and SDF-1α. Therefore, our results demonstrate that the inhibition of cytokine storm may be a potential mechanism by which FFYH can treat COVID-19. Additionally, it has been reported that elevated IL-6 levels are significantly associated with severity in patients with COVID-19 (Zizzo et al., 2022). Our results revealed that FFYH treatment had a greater inhibitory effect on IL-6 expression than LHQW treatment (*P* < 0.05). In contrast, compared with that in the pretreatment group, the expression level of IL-6 in the LHQW treatment group was not significantly greater, which is consistent with the findings of Shen et al., who reported that LHQW had no inhibitory effect on IL-6 expression in COVID-19 patients (Shen et al., 2021). Furthermore, our previous work also indicated that FFYH treatment markedly inhibited the expression of IL-6 in influenza virus pneumonia (Zhang et al., 2021). Therefore, we speculated that FFYH may be more effective for COVID-19 patients with abnormally elevated IL-6 levels.

It has been reported that the PI3K/AKT/mTOR signaling pathway can regulate the secretion of inflammatory cytokines by T cells. However, inhibiting this pathway may be a potential therapeutic strategy when COVID-19 patients progress to uncontrolled cytokine storms (Abu-Eid and Ward, 2021). On the basis of the *in vitro* study results, we hypothesize that the mechanism by which FFYH inhibits cytokine storms may be related to its ability to suppress the PI3K/AKT/mTOR signaling pathway. However, future work is needed to better understand the underlying mechanism of the effects of FFYH against SARS-CoV-2 through the regulation of the PI3K/AKT/mTOR signaling pathway.

## Limitations

This study has several limitations. First, since the patients were recruited from a single hospital, the therapeutic effect of FFYH on COVID-19 needs to be further verified by large-cohort randomized controlled clinical trials. Second, owing to the lack of a placebo and positive anti-viral drug control, we were unable to compare the alleviating effects of FFYH on COVID-19 symptoms with those of other drugs. Third, most patients were discharged 5–6 days after treatment because of significantly improved symptoms, and we could not collect sufficient data on the conversion rate of nucleic acid negativity.

## Conclusion

In summary, our results indicate that FFYH can effectively inhibit the replication of SARS-CoV-2 by blocking autophagy via the PI3K/AKT/mTOR signaling pathway and has a significant therapeutic effect by improving clinical symptoms, restoring hematological parameters, preventing disease progression, shortening the duration of hospitalization, and controlling excessive cytokine storms in mild COVID-19 patients, suggesting that FFYH may be a promising option for treating COVID-19.

## Data Availability

The original contributions presented in the study are included in the article/Supplementary Material; further inquiries can be directed to the corresponding authors.
