# Supplementary material for: Clinical efficacy of Fufang Yinhua Jiedu (FFYH) granules in mild COVID-19 and its anti-SARS-CoV-2 mechanism by blocking autophagy through inhibiting the AKT/mTOR signaling pathway
Source: Front Pharmacol. 2024 Sep 16;15:1431617. doi: 10.3389/fphar.2024.1431617 (PMC11439717; doi:10.3389/fphar.2024.1431617)
Supplement: Supplementary file 1 [file Table1.DOCX]

Table S1. Improvement of clinical symptoms in COVID-19 patients treated with FFYH or LHQW

| Term | FFYH (N=99) | LHQW (N=100) | *P*-value |
| --- | --- | --- | --- |
| Clinical symptoms recovery (%) | 41 (41.4) | 33 (33.0) | 0.1869 |
| Time to clinical symptoms recovery (d, M±SD) | 5.7±1.7 | 6.0±1.3 |  |
| Fever (%) | 47 (100.0) | 36 (97.3) | 0.7491 |
| Time to fever recovery (h, M±SD) | 46.8±37.0 | 45.5±38.9 |  |
| Respiratory symptoms recovery (%) | 42 (44.7) | 32 (33.0) | 0.0951 |
| Time to respiratory symptoms recovery (d, M±SD) | 5.7±1.6 | 6.0±1.4 |  |
| Fatigue (%) | 54 (77.1) | 61 (87.1) | 0.2170 |
| Time to fatigue recovery (d, M±SD) | 3.2±1.6 | 3.2±1.9 |  |
| Cough (%) | 38 (47.5) | 33 (39.3) | 0.2263 |
| Time to cough recovery (d, M±SD) | 4.5±2.1 | 4.5±2.1 |  |
| Dried pharynx (%) | 57 (70.4) | 57 (77.0) | 0.4288 |
| Time to dried pharynx recovery (d, M±SD) | 4.1±1.9 | 4.0±1.9 |  |
| Sore throat (%) | 57 (96.4) | 42 (80.8) | 0.4320 |
| Time to sore throat recovery (d, M±SD) | 3.9±2.0 | 3.9±2.0 |  |
| Nasal congestion (%) | 31 (72.1) | 41 (77.4) | 0.8151 |
| Time to nasal congestion recovery (d, M±SD) | 3.7±2.1 | 4.2±2.1 |  |
| Runny nose (%) | 30 (73.2) | 32 (74.4) | 0.5837 |
| Time to runny nose recovery (d, M±SD) | 4.3±1.9 | 3.8±2.2 |  |
| Taste disorder (%) | 26 (86.7) | 18 (94.7) | 0.1680 |
| Time to taste disorder recovery (d, M±SD) | 3.3±2.1 | 2.5±1.6 |  |
| Olfactory disorder (%) | 5 (83.3) | 5 (83.3) | 0.4328 |
| Time to olfactory disorder recovery (d, M±SD) | 2.5±1.9 | 3.3±2.4 |  |
| Other symptoms (%) | 8 (88.9) | 9 (81.8) | 0.3183 |
| Time to other symptoms recovery (d, M±SD) | 2.5±1.6 | 3.0±1.8 |  |

Table S2. Improvement of clinical symptoms in male COVID-19 patients treated with FFYH or LHQW

| Term | FFYH (N=48) | LHQW  (N=53) | *P*-value |
| --- | --- | --- | --- |
| Clinical symptoms recovery (%) | 26 (54.2) | 18 (34.0) | 0.0349^*^ |
| Time to clinical symptoms recovery (d, M±SD) | 5.3±1.9 | 5.8±1.5 |  |
| Fever (%) | 21 (100.0) | 15 (100.0) | 0.5080 |
| Time to fever recovery (h, M±SD) | 43.0±34.7 | 50.1±42.7 |  |
| Respiratory symptoms recovery (%) | 25 (56.8) | 18 (34.6) | 0.0234^*^ |
| Time to respiratory symptoms recovery (d, M±SD) | 5.3±1.8 | 5.8±1.6 |  |
| Fatigue (%) | 29 (93.5) | 29 (87.9) | 0.3240 |
| Time to fatigue recovery (d, M±SD) | 2.9±1.7 | 3.2±1.8 |  |
| Cough (%) | 20 (57.1) | 19 (45.2) | 0.4755 |
| Time to cough recovery (d, M±SD) | 4.6±1.9 | 4.1±2.1 |  |
| Dried pharynx (%) | 28 (77.8) | 33 (91.7) | 0.2622 |
| Time to dried pharynx recovery (d, M±SD) | 3.8±1.8 | 3.9±1.9 |  |
| Sore throat (%) | 24 (92.3) | 21 (87.5) | 0.7769 |
| Time to sore throat recovery (d, M±SD) | 3.9±2.2 | 4.0±2.1 |  |
| Nasal congestion (%) | 17 (85.0) | 25 (83.3) | 0.2267 |
| Time to nasal congestion recovery (d, M±SD) | 3.4±2.1 | 4.3±2.0 |  |
| Runny nose (%) | 13 (72.2) | 17 (73.9) | 0.8082 |
| Time to runny nose recovery (d, M±SD) | 4.1±1.9 | 3.7±2.1 |  |
| Taste disorder (%) | 13 (100.0) | 8 (100.0) | 0.3115 |
| Time to taste disorder recovery (d, M±SD) | 3.0±1.9 | 2.1±1.7 |  |
| Olfactory disorder (%) | 2 (100.0) | 2 (100.0) | 0.5351 |
| Time to olfactory disorder recovery (d, M±SD) | 1.7±1.2 | 3.0±3.5 |  |
| Other symptoms (%) | 5 (100.0) | 5 (83.3) | 0.2991 |
| Time to other symptoms recovery (d, M±SD) | 2.0±1.7 | 2.6±1.8 |  |

^*^*P*<0.05, FFYH VS LHQW.

Table S3. Improvement of clinical symptoms in female COVID-19 patients treated with FFYH or LHQW

| Term | FFYH (N=51) | LHQW  (N=47) | P-value |
| --- | --- | --- | --- |
| Clinical symptoms recovery (%) | 15 (29.4) | 15 (31.9) | 0.7851 |
| Time to clinical symptoms recovery (d, M±SD) | 6.3±1.0 | 6.3±1.1 |  |
| Fever (%) | 26 (100.0) | 21 (95.5) | 0.7201 |
| Time to fever recovery (h, M±SD) | 49.9±39.1 | 42.1±36.5 |  |
| Respiratory symptoms recovery (%) | 17 (34.0) | 14 (31.1) | 0.8508 |
| Time to respiratory symptoms recovery (d, M±SD) | 6.3±1.0 | 6.3±1.1 |  |
| Fatigue (%) | 25 (64.1) | 32 (86.5) | 0.0278^*^ |
| Time to fatigue recovery (d, M±SD) | 3.5±1.5 | 3.1±2.0 |  |
| Cough (%) | 18 (40.0) | 14 (33.3) | 0.2234 |
| Time to cough recovery (d, M±SD) | 4.5±2.3 | 5.1±2.0 |  |
| Dried pharynx (%) | 29 (64.4) | 24 (63.2) | 0.9609 |
| Time to dried pharynx recovery (d, M±SD) | 4.5±2.0 | 4.1±1.9 |  |
| Sore throat (%) | 33 (82.5) | 21 (75.0) | 0.3938 |
| Time to sore throat recovery (d, M±SD) | 3.9±1.9 | 3.7±1.8 |  |
| Nasal congestion (%) | 14 (60.9) | 16 (69.6) | 0.6202 |
| Time to nasal congestion recovery (d, M±SD) | 4.1±2.0 | 4.1±2.2 |  |
| Runny nose (%) | 17 (73.9) | 15 (75.0) | 0.6224 |
| Time to runny nose recovery (d, M±SD) | 4.4±2.0 | 3.9±2.3 |  |
| Taste disorder (%) | 13 (76.5) | 10 (90.9) | 0.2554 |
| Time to taste disorder recovery (d, M±SD) | 3.7±2.3 | 2.7±1.6 |  |
| Olfactory disorder (%) | 3 (75.0) | 3 (75.0) | 0.7542 |
| Time to olfactory disorder recovery (d, M±SD) | 3.3±2.3 | 3.5±1.7 |  |
| Other symptoms (%) | 3 (75.0) | 4 (80.0) | 0.6195 |
| Time to other symptoms recovery (d, M±SD) | 3.0±1.6 | 3.5±1.9 |  |

^*^*P*<0.05, FFYH VS LHQW.

Table S4. Improvement of clinical symptoms in COVID-19 patients with baseline hematology abnormalities treated with FFYH or LHQW

| Term | FFYH (N=87) | LHQW  (N=85) | *P*-value |
| --- | --- | --- | --- |
| Clinical symptoms recovery (%) | 38 (43.7) | 24 (28.2) | 0.0140^*^ |
| Time to clinical symptoms recovery (d, M±SD) | 5.7±1.6 | 6.3±1.1 |  |
| Fever (%) | 44 (100.0) | 32 (97.0) | 0.5821 |
| Time to fever recovery (h, M±SD) | 47.8±36.5 | 47.9±40.1 |  |
| Respiratory symptoms recovery (%) | 38 (46.3) | 23 (28.0) | 0.0055^**^ |
| Time to respiratory symptoms recovery (d, M±SD) | 5.7±1.6 | 6.4±1.1 |  |
| Fatigue (%) | 46 (76.7) | 52 (85.2) | 0.4601 |
| Time to fatigue recovery (d, M±SD) | 3.1±1.7 | 3.3±1.9 |  |
| Cough (%) | 34 (50.0) | 25 (35.2) | 0.0352^*^ |
| Time to cough recovery (d, M±SD) | 4.6±2.1 | 4.8±2.1 |  |
| Dried pharynx (%) | 51 (73.9) | 48 (75.0) | 0.9348 |
| Time to dried pharynx recovery (d, M±SD) | 4.1±1.9 | 4.1±2.0 |  |
| Sore throat (%) | 47 (85.5) | 37 (78.7) | 0.1744 |
| Time to sore throat recovery (d, M±SD) | 3.8±2.0 | 4.0±2.0 |  |
| Nasal congestion (%) | 26 (76.5) | 30 (73.2) | 0.1356 |
| Time to nasal congestion recovery (d, M±SD) | 3.6±2.1 | 4.5±2.2 |  |
| Runny nose (%) | 25 (75.8) | 25 (73.5) | 0.9696 |
| Time to runny nose recovery (d, M±SD) | 4.2±1.9 | 3.9±2.2 |  |
| Taste disorder (%) | 24 (88.9) | 13 (92.9) | 0.2098 |
| Time to taste disorder recovery (d, M±SD) | 3.3±2.1 | 2.1±1.3 |  |
| Olfactory disorder (%) | 5 (83.3) | 5 (83.3) | 0.4328 |
| Time to olfactory disorder recovery (d, M±SD) | 2.5±1.9 | 3.3±2.4 |  |
| Other symptoms (%) | 7 (87.5) | 7 (77.8) | 0.1444 |
| Time to other symptoms recovery (d, M±SD) | 2.1±1.5 | 3.4±1.8 |  |

^*^*P*<0.05, ^**^*P*<0.01, FFYH VS LHQW.

Table S5. Improvement of clinical symptoms in COVID-19 patients aged 33~42 years treated with FFYH or LHQW

| Term | FFYH  (N=29) | LHQW  (N=33) | P-value |
| --- | --- | --- | --- |
| Clinical symptoms recovery (%) | 11 (37.9) | 5 (15.2) | 0.0517 |
| Fever (%) | 13 (100.0) | 10 (90.9) | 0.4204 |
| Respiratory symptoms recovery (%) | 11 (40.7) | 5 (15.6) | 0.0387^*^ |
| Fatigue (%) | 16 (80.0) | 21 (91.3) | 0.5069 |
| Cough (%) | 10 (43.5) | 6 (23.1) | 0.0617 |
| Dried pharynx (%) | 21 (77.8) | 20 (76.9) | 0.6681 |
| Sore throat (%) | 21 (91.3) | 13 (76.5) | 0.0345^*^ |
| Nasal congestion (%) | 9 (75.0) | 15 (83.3) | 0.8371 |
| Runny nose (%) | 6 (75.0) | 15 (83.3) | 0.7802 |
| Taste disorder (%) | 8 (72.7) | 7 (100.0) | 0.1171 |
| Olfactory disorder (%) | 1 (100.0) | 2 (100.0) | 0.0833 |
| Other symptoms (%) | 2 (100.0) | 3 (75.0) | 0.1138 |

^*^*P*<0.05 FFYH VS LHQW.

Table S6. Improvement of clinical symptoms in COVID-19 patients aged 18~32 years treated with FFYH or LHQW

| Term | FFYH  (N=31) | LHQW  (N=36) | P-value |
| --- | --- | --- | --- |
| Clinical symptoms recovery (%) | 14 (45.2) | 13 (36.1) | 0.3048 |
| Fever (%) | 18 (100.0) | 16 (100.0) | 0.4030 |
| Respiratory symptoms recovery (%) | 15 (48.4) | 13 (36.1) | 0.2097 |
| Fatigue (%) | 18 (78.3) | 23 (85.2) | 0.7932 |
| Cough (%) | 14 (48.3) | 15 (44.1) | 0.6920 |
| Dried pharynx (%) | 19 (76.0) | 20 (76.9) | 0.5335 |
| Sore throat (%) | 19 (90.5) | 18 (85.7) | 0.2845 |
| Nasal congestion (%) | 14 (82.4) | 17 (73.9) | 0.2477 |
| Runny nose (%) | 17 (94.4) | 12 (66.7) | 0.0709 |
| Taste disorder (%) | 11 (91.7) | 7 (100.0) | 0.0558 |
| Olfactory disorder (%) | 2 (66.7) | 2 (100.0) | 0.9945 |
| Other symptoms (%) | 3 (100.0) | 5 (100.0) | 0.1117 |

Table S7. Improvement of clinical symptoms in COVID-19 patients older than 42 years treated with FFYH or LHQW

| Term | FFYH  (N=39) | LHQW  (N=31) | P-value |
| --- | --- | --- | --- |
| Clinical symptoms recovery (%) | 16 (41.0) | 15 (48.4) | 0.5349 |
| Fever (%) | 16 (100.0) | 10 (100.0) | 0.0910 |
| Respiratory symptoms recovery (%) | 16 (44.4) | 14 (48.3) | 0.6973 |
| Fatigue (%) | 20 (74.1) | 17 (85.0) | 0.0985 |
| Cough (%) | 14 (50.0) | 12 (50.0) | 0.7633 |
| Dried pharynx (%) | 17 (58.6) | 17 (77.3) | 0.2065 |
| Sore throat (%) | 17 (77.3) | 11 (78.6) | 0.1444 |
| Nasal congestion (%) | 8 (57.1) | 9 (75.0) | 0.3687 |
| Runny nose (%) | 7 (46.7) | 5 (71.4) | 0.0854 |
| Taste disorder (%) | 7 (100.0) | 4 (80.0) | 0.5090 |
| Olfactory disorder (%) | 2 (100.0) | 1 (50.0) | 0.5127 |
| Other symptoms (%) | 3 (75.0) | 1 (50.0) | 0.7540 |
