# Supplementary material for: Clinical efficacy of Fufang Yinhua Jiedu (FFYH) granules in mild COVID-19 and its anti-SARS-CoV-2 mechanism by blocking autophagy through inhibiting the AKT/mTOR signaling pathway
Source: Front Pharmacol. 2024 Sep 16;15:1431617. doi: 10.3389/fphar.2024.1431617 (PMC11439717; doi:10.3389/fphar.2024.1431617)
Supplement: Supplementary file 2 [file Table2.docx]

**Supplementary materials 2**

**MATERIALS AND METHODS**

**Preparation process of FFYH**

The preparation process of FFYH is as follows: extract of volatile oils from *Artemisia annua* L, *Lonicerae japonica* Thunb, *Schizonepeta tenuifolia* (Benth.) Briq, *Chrysanthemum indicum* L, *Forsythia suspensa* (Thunb.) Vahl, and *Peucedanum praeruptorum* Dunn. Store the post-distillation aqueous solution separately. The remaining medicinal residues, along with other ingredients, are decocted twice with 9 times the amount of water, each time for 1.5 hours. Combine the decoctions with the distilled liquid, filter, and concentrate the filtrate to a relative density of 1.15~1.20 (80°C). Add ethanol to achieve an alcohol content of 63%, stir well, let it stand, and recover the ethanol from the supernatant, then concentrate to a relative density of 1.25~1.30 (80°C). Take one part of the extract, mix with three parts of sucrose, one part dextrin, and an appropriate amount of ethanol to form granules, then dry to obtain 1000g. Add the previously extracted volatile oils of *Artemisia annua* L and other ingredients, mix well to obtain the final product. Pharmaceutical Standard Number: YBZ00602004.

**Quality control of FFYH**

The main components of FFYH were analyzed by HPLC using Kromasil 100-5-C18 (4.6 mm× 250 mm, 5 μm) as the packing material. The mobile phase consisted of acetonitrile (A) and 0.1% phosphoric acid (B), at a flow rate of 1.0 mL/min. The detection wavelength was set at 230 nm, with the elution gradient set in Table S8, and the column temperature maintained at 30°C.

Table S8 Elution Gradient Settings for HPLC Analysis of FFYH

| **Time (min)** | **mobile phase A (%)** | **mobile phase B (%)** |
| --- | --- | --- |
| 0～10 | 5→10 | 95→90 |
| 10～25 | 10→12 | 90→88 |
| 25～50 | 12→22 | 88→78 |
| 50～60 | 22→32 | 78→68 |
| 60～65 | 32→40 | 68→60 |
| 65～70 | 40→85 | 60→15 |
| 70～80 | 85→95 | 15→5 |

nd 0.4%, respectively.
